# Prediction of Gastric Residual Volume by Ultrasonography in Critically Ill Children Undergoing Enteral Nutrition

**DOI:** 10.1155/ccrp/1049746

**Published:** 2025-06-23

**Authors:** Jinjiu Hu, Qiaoying Zhang, Xin Wan, Hui Zhang, Qiao Shen, Fei Li, Ye Cai, Yuqian Meng, Peng Liu, Xianlan Zheng

**Affiliations:** ^1^Department of Nursing, Children's Hospital of Chongqing Medical University, National Clinical Research Center for Child Health and Disorders, Chongqing Key Laboratory of Pediatric Metabolism and Inflammatory Diseases, Ministry of Education Key Laboratory of Child Development and Disorders, Chongqing, China; ^2^Pediatric Intensive Care Unit, Children's Hospital of Chongqing Medical University, Chongqing, China

**Keywords:** child, enteral nutrition, gastric residual volume, pediatric intensive care unit, ultrasonography

## Abstract

**Background:** Bedside ultrasonography is capable of evaluating gastric residual volume (GRV) and facilitating the identification of feeding intolerance (FI) among critically ill pediatric patients; however, a specialized predictive model tailored to this demographic has yet to be established. This study aims to develop a predictive model for the estimation of GRV using ultrasonography in this specific patient group.

**Methods:** This prospective observational study included critically ill pediatric patients receiving enteral nutrition (EN). Clinical data, including gender, age, weight, height, gastric antrum cross-sectional area (CSA) in supine and right lateral positions, and qualitative grading system scores (Grade 0–2), were collected. GRV was measured by suctioning gastric contents under real-time ultrasound guidance, which was considered the actual GRV. The predictive models for GRV were developed using linear regression analysis. The agreement between predicted and actual GRV values was assessed using Bland–Altman analysis.

**Results:** A total of 108 children were included in the analysis. Significant differences (*p* < 0.05) were observed in GRV, GRV per kilogram, supine and right lateral decubitus (RLD) CSA among grades. Spearman correlation analysis revealed strong correlations between RLD CSA (*r* = 0.88, *p* < 0.001) and qualitative grading system scores (*r* = 0.86, *p* < 0.001) with suctioned GRV. A predictive model was developed using RLD CSA and qualitative grading system scores as predictors: GRV (mL) = −12.9 + 10.3 (RLD CSA [cm^2^]) + 3.3 × Grade 1 + 10.1 × Grade 2. This model demonstrated an adjusted coefficient of determination (*R*^2^) of 0.878, Akaike's information criterion (AIC) of 873.43, and Bayesian information criterion (BIC) of 884.06. Bland–Altman analysis showed a mean difference of 0.1 mL/kg between predicted and suctioned GRV, with 95% limits of agreement (LoA) ranging from −1.65 to 1.87 mL/kg.

**Conclusion:** The results suggest that ultrasound-based monitoring can predict GRV in critically ill children. In addition, the qualitative grading system can differentiate between high and low GRV, potentially serving as a rapid screening tool for identifying patients with high GRV.

## 1. Introduction

Children in pediatric intensive care units (PICUs) are at increased risk of malnutrition [1]. Poor nutritional status is associated with several adverse clinical outcomes, including prolonged mechanical ventilation, muscle wasting, impaired immune function, increased susceptibility to infections, and higher morbidity and mortality rates [2–4]. Enteral nutrition (EN) is the preferred support for critically ill children, offering benefits such as preserving intestinal mucosal barrier function, promoting gut microbiota stability, and enhancing immunity [5]. However, factors such as underlying diseases, analgesic/sedative usage, and surgeries heighten feeding intolerance (FI) risks [6], manifested by high gastric residual volume (GRV), vomiting, abdominal distension, and diarrhea [7].

Currently, gastric tube suction is commonly used to assess GRV clinically, but its accuracy is affected by patient positioning, tube composition, and other factors [8]. There is also a risk of contamination during suction/reinjection, and the discard of gastric contents can lead to the loss of gastric acid, pepsin, electrolytes, etc. [9]. Recent studies indicate that ultrasound can effectively evaluate GRV both qualitatively and quantitatively, with results effectively reflecting the state or volume of stomach contents [10, 11]. This makes it a feasible and promising tool for GRV monitoring in clinical practice [12]. Its principle is measuring gastric antrum cross-sectional area (CSA) via ultrasound and calculating GRV using models.

Previous pediatric GRV prediction formulas were developed based on preoperatively fasted children. Kim et al. examined 200 preoperatively fasted children, of those, 84.3% children had empty stomachs, and over half of Spencer et al.'s 100-subject cohort showed empty stomachs [13, 14], which suggests that fasting children tend to have empty stomachs. Conversely, children with high GRV are more likely to be observed in the PICU, with an incidence rate of 22% [15]. Moreover, the threshold of high GRV typically was set at 50% of the last feeding volume, so the proportion of nonempty stomachs is higher in this population [16]. This population heterogeneity may cause previous formulas to underestimate GRV in critically ill children.

In addition, a semiquantitative three-point qualitative grading system was developed to differentiate between low- and high-volume states [17]. This system is based on a qualitative assessment of the clear fluid-containing gastric antrum in supine and right lateral decubitus (RLD) positions. The grades are defined as follows: Grade 0 (no fluid visible in either position), Grade 1 (fluid visible only in the RLD), and Grade 2 (fluid visible in both positions). This system has been validated in preoperatively fasted adults and children and is considered a rapid screening tool for perioperative aspiration risk [14, 17].

Therefore, this prospective study aimed to develop a predictive model for GRV in critically ill children receiving EN using a previously published qualitative grading system and other potential factors.

## 2. Methods

### 2.1. Study Design and Setting

This prospective observational study was approved by the Institutional Review Board of the Children's Hospital of Chongqing Medical University (No: 2024.475; Approval date: January 3, 2025). Informed written consent was obtained from legal guardians or patients capable of understanding the study. Participants were recruited through stratified convenience sampling, with children categorized into five age groups: 1 month–1 year, 1–3 years, 3–6 years, 6–12 years, and 12–18 years. Inclusion criteria were children aged 1 month to 18 years and receiving EN via a gastric tube. Exclusion criteria were impaired gastrointestinal function (e.g., severe gastrointestinal dysfunction and history of gastrointestinal surgery), conditions affecting monitoring results (e.g., bloating, obesity, and abdominal wall abnormalities), inability to change position (e.g., intracranial hemorrhage and spinal injuries), and infectious diseases. The study period was from February 13, 2025, to March 15, 2025.

### 2.2. Variables and Assessment

Demographic data (gender, age, weight, height, and admission diagnosis) were extracted from electronic medical records. Gastric ultrasound assessments were performed by two certified physicians or nurses from the Chinese Critical Care Ultrasound Study Group (CCCUSG). One operator positioned the child supine with the bed elevated at 45° and used a portable color diagnostic ultrasound device with a convex array probe (2–5 Hz) or linear probe (5–12 Hz). The probe was placed vertically on the abdomen beneath the xiphoid process, and a single-sectional scan of the gastric antrum was performed. The gastric antrum is situated near the superior mesenteric artery, the left lobe of the liver, and the abdominal aorta. A CSA was obtained in the supine position. Subsequently, the child was repositioned to the right lateral recumbent position, and the CSA in this orientation was measured. Meanwhile, the other operator aspirates the gastric contents using a 50 mL syringe under real-time ultrasound monitoring until the stomach appears empty. Monitoring is performed within 10 min prior to each feeding.

At least one video clip and three still images of the gastric antrum were obtained in both positions. Only one researcher was responsible for measuring the gastric antrum CSA using the tracing method to delineate the outer gastric wall (plasma membrane layer). The final CSA was the average of three measurements. GRV also was assessed using a qualitative grading system: Grade 0 (no fluid visible in either position), Grade 1 (fluid visible only in the right lateral position), and Grade 2 (fluid visible in both positions). An empty stomach was characterized by a flattened gastric antrum with the “target sign” or “bull's eye sign,” while a nonempty stomach had a cavity between the anterior and posterior walls. During monitoring, the operator monitored the child's vital signs and collaborated with the attending physician and nurse to deal with any changes promptly. In addition, the ultrasound probe and machine surfaces were wiped with disinfectant wipes before and after monitoring.

### 2.3. Sample Size

Based on previous research, seven potential predictors were identified for regression analysis: gender, age, weight, height, CSA both in supine and RLD, and qualitative grading system scores. We adhered to the events as per the variable criterion, which recommends a minimum of 10 observations per predictor variable to ensure model stability [18]. Accounting for an anticipated 10% attrition rate, the total target enrollment was set at 77 children. Given the physiological differences across pediatric ages [19], participants were systematically allocated into five age cohorts: 1 month–1 year, 1–3 years, 3–6 years, 6–12 years, and 12–18 years. Each cohort targeted 16 participants. Given the above two aims, the total target enrollment was set at 80 children.

### 2.4. Statistical Analysis

The Kolmogorov–Smirnov test was used to test the normality of the data distribution. Data with normal distribution were statistically described by mean ± standard deviation, and non-normally distributed data or counts were described by median (interquartile spacing). Correlations between suctioned GRV and independent variables were characterized using the Spearman correlation coefficient. Based on the results of correlation analysis and previous studies, the relevant variables were decided to be included in linear regression to construct the prediction model of GRV. Finally, Bland–Altman analysis was used for consistency analysis between the predicted GRV per kilogram and the suctioned GRV per kilogram. All statistical tests were completed using IBM SPSS Statistics 27 software (IBM, Armonk NY, USA).

## 3. Result

A total of 113 children were included in this study. After adjustments to the depth and position of the gastric tube, five children were excluded due to the inability to aspirate gastric contents, despite the presence of gastric contents on ultrasound. The remaining 108 children were included in the analysis.

Demographic and clinical characteristics of the children are presented in Table 1. Of the 108 children, 68 (63%) were male. The median age was 2.62 years (interquartile range [IQR]: 0.55, 6.87), median weight was 13 kg (IQR: 6.90, 25.00), and median height was 0.9 m (IQR: 0.66, 1.28). Stomach contents were observed in 65.7% of children, with 22.2% showing Grade 1, 43.5% showing Grade 2, and 34.3% showing no detectable contents (Grade 0). Upon admission, diagnoses included respiratory disease in 40 (37.0%), craniocerebral injury in 31 (28.7%), circulatory disease in 6 (5.6%), neurological disease in 4 (3.7%), and shock in 3 (2.8%) children. No solid materials were observed.

This study compared median GRV, GRV per kilogram, and CSA in both supine and RLD among grades (Table 2). The median GRV per kilogram for Grades 0, 1, and 2 were 0, 1.29, and 2.22 mL/kg, respectively. Statistically significant differences were observed among groups (*p* < 0.05). Median supine CSA values were 0.95, 1.07, and 2.49 cm^2^, while median RLD CSA values were 1.27, 1.98, and 4.55 cm^2^. Both CSA measures showed significant differences across groups (*p* < 0.05).

Spearman correlation analysis revealed positive correlations between GRV and age (*r* = 0.34, *p* < 0.05), weight (*r* = 0.34, *p* < 0.05), height (*r* = 0.35, *p* < 0.05), BMI (*r* = 0.061, *p* < 0.05), supine CSA (*r* = 0.63, *p* < 0.05), RLD CSA (*r* = 0.88, *p* < 0.05), and qualitative grading system scores (*r* = 0.87, *p* < 0.05). The RLD CSA and qualitative grading system scores showed the strongest correlations (Figure Supporting 1).

Based on the above results and previous studies, age, RLD CSA, and qualitative grading system scores were included in linear regression models. Dummy variables were created with “Grade 0” as the reference: Grade 1 = (1, 0) and Grade 2 = (0, 1).  Table 3 shows that RLD CSA is an independent predictor of GRV. Model 1: GRV (mL) = −13 + 0.06 × age + 10.3 (RLD CSA [cm^2^]) + 3.4 × Grade 1 + 10.1 × Grade 2 (adjusted coefficient of determination (*R*^2^) = 0.877, Akaike information criterion [AIC] = 875.30, and Bayesian information criterion [BIC] = 888.71). Model 2, excluding age, showed superior performance: GRV (mL) = −12.9 + 10.3 (RLD CSA [cm^2^]) + 3.3 × Grade 1 + 10.1 × Grade 2 (adjusted *R*^2^ = 0.878, AIC = 873.43, and BIC = 884.06). Model 2 was selected due to its higher adjusted *R*^2^ and lower AIC/BIC values, and fewer variables, enhancing clinical applicability. Note that, positive values are meaningful and negative values are considered empty stomachs in our model.

**Figure 1:**
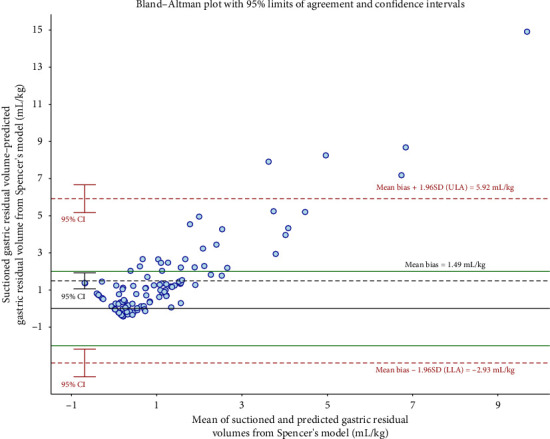
Bland–Altman analysis plots the difference between predicted (Spencer model) and suctioned gastric residual volume for all children versus the mean volume of the predicted and suctioned values. LLA: lower limit of agreement; ULA: upper limit of agreement; CI: confidence interval; SD: standard deviation; Spencer formula: GRV (mL) = −7.8 + 3.5 [RLD CSA] + 0.127 × age (months).

**Figure 2:**
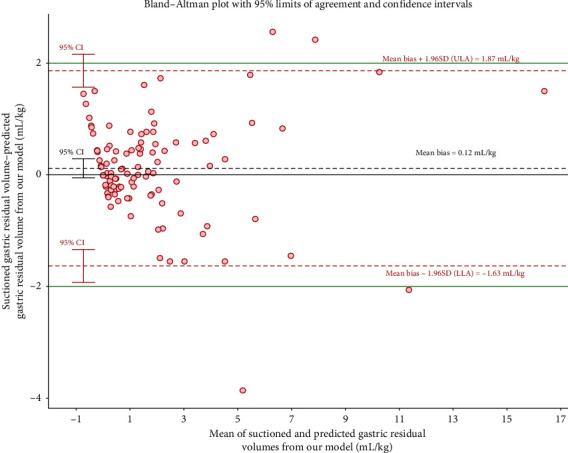
Bland–Altman analysis plots the difference between predicted (our formula) and suctioned gastric residual volume for all children versus the mean volume of the predicted and suctioned values. LLA: lower limit of agreement; ULA: upper limit of agreement; CI: confidence interval; SD: standard deviation; our formula: GRV (mL) = −12.9 + 10.3 [RLD CSA (cm^2^)] + 3.3 × Grade 1 + 10.1 × Grade 2.

Bland–Altman analysis was used to assess the agreement between the predicted GRV per kilogram and the suctioned GRV per kilogram.  Figure 1 shows that the mean difference between the predicted GRV per kilogram and the suctioned GRV per kilogram using Spencer et al.'s model was 1.49 mL/kg, with 95% limits of agreement (95% LoA) ranging from −2.93 to 5.92 mL/kg, and 4.6% of data points falling outside the 95% LoA.  Figure 2 shows that the mean difference between the predicted GRV per kilogram and the suctioned GRV per kilogram using our model was 0.11 mL/kg, with 95% LoA ranging from −1.63 to 1.86 mL/kg, and 5.5% of data points falling outside the 95% LoA. The clinically acceptable limits were ±2 mL/kg. The mean difference line in Figure 2 is closer to the zero line than that in Figure 1, indicating greater consistency between that predicted from our model and actual GRV. The scatterplot (Figure supporting 2) shows the distribution of GRV predictions from both models.

## 4. Discussion

This study validated the qualitative grading system's applicability for assessing GRV in critically ill children receiving EN. We developed a predictive model to estimate GRV using the child's right lateral CSA and the qualitative grading system scores.

Correlation analysis revealed a 0.63 correlation between suctioned GRV and supine CSA, consistent with Spencer (*r* = 0.63) and Kim (*r* = 0.667) [13, 14]. The correlation between suctioned GRV and RLD CSA was 0.88, aligning with Kim's findings (*r* = 0.845) but exceeding Spencer's (*r* = 0.67) [13, 14]. Combined with previous studies, we found a stronger association between suctioned GRV and RLD CSA compared to the supine position. In addition, CSA is also affected by the angle of bed head elevation. Bouvet et al. [20] examined ultrasound images of fasting children with head elevation angles of 0°, 30°, and 45° after drinking water and found that GRV was more easily detected at 45° elevation. This suggests that CSA is influenced by body position, as gastric contents are more likely to move toward the distal gastric region (gastric antrum) due to gravity in the right lateral position and supine (with the bed head elevated at 45°) [21]. The correlation between suctioned GRV and qualitative grading system scores in this study (*r* = 0.86) exceeded Kim's findings (*r* = 0.581) [13]. This discrepancy may be due to the higher proportion of preoperatively fasted children with empty stomachs in Kim's study.

In fact, some pediatric GRV ultrasound prediction models have been developed, but these models are not applicable to critically ill children and have a small sample size. Schmitz et al. [22] used ultrasound to measure the GRV in 16 children aged 6–13 years in the RLD, supine positions, with magnetic resonance imaging as the reference standard. They derived the following formula: GRV (mL) = 0.0093 (RLD CSA [mm^2^]) − 1.36 (*R*^2^ = 0.6). This predictive model is the first study to use magnetic resonance imaging to assess GRV, but the error between the predicted and actual measured values reached 2.8 mL/kg. Gagey et al. [23] compared data from 34 neonates with hypertrophic pyloric stenosis at 4–6 weeks of age with preoperative GRV and ultrasound assessment of the CSA of the gastric antrum in the RLD and derived the formula: GRV (ml) = 1.42 + 4.18 (RLD CSA [cm^2^]) − 1.24 × age (weeks) − 0.65 × weight (kg) (*R*^2^ = 0.69). However, this population is in a state of gastric dysfunction, with limited clinical guidance.

This study constructed a GRV model using related factors of critically ill children and selected Model 2 (after calling our model or our formula) based on *R*^2^, AIC, and BIC as the optimal model. Our model achieved an adjusted *R*^2^ of 0.878, which surpassed the Spencer formula [14]. Consistent with prior studies, RLD CSA was identified as an independent predictor of GRV [13, 14]. However, in contrast to Spencer et al.'s findings, age was not an independent predictor of GRV in our cohort. In Spencer's study, 54% of participants had empty stomachs, which were characterized by closely apposed gastric walls [24]. The age-related increase in gastric wall thickness suggests that older children with empty stomachs may have larger CSA [19]. Instead, in the present study, only 34.3% of children had empty stomachs. The remaining children had more gastric contents that thinned the gastric wall, which reduced the predictive value of age for CSA, which could explain this discrepancy. Lin's findings seem to support our view, as GRV levels reflect intestinal tolerance, but age is not an independent predictor of FI in critically ill children [25].

Bland–Altman analysis was used to assess the agreement between model-predicted GRV per kilogram and suctioned GRV per kilogram. The Spencer et al.'s model showed a larger mean difference and a trend of increasing discrepancy with higher suctioned GRV compared to our model (Figures 1 and 2). Scatterplot data show both models perform similarly at lower GRV, but differences become greater at higher GRV. This finding may be attributed to the heterogeneity between fasting children and critically ill children receiving EN.

Critically ill pediatric patients often experience FI during EN, with high GRV being a primary reason [26]. Healthcare professionals typically recommend suspending feeding in response to high GRV, which can lead to unnecessary EN interruptions and result in insufficient energy intake [27, 28]. Owing to its simplicity and cost-effectiveness, suctioning gastric tubes is the most frequently used method for monitoring GRV in clinical settings. However, frequent use of empty needle aspiration may waste nursing time and increase consumables, while not re-injecting gastric contents may lead to the loss of gastric acid and proteases [9]. Researchers have used ultrasound to monitor GRV and guide EN in critically ill adults, which showed some benefits [29, 30]. However, current international guidelines do not recommend routine GRV monitoring in intensive care units as it does not reduce aspiration and pneumonia risks but may lead to unnecessary feeding interruptions and malnutrition [5, 31].However, a recent study revealed variations in the interpretation of FI, GRV threshold, and management approaches across units, despite these recommendations [32].

## 5. Limitations

This study has several limitations. First, the predictive model lacks external validation. Future studies with larger sample sizes are needed to refine the model. Second, suctioned GRV was defined as actual GRV, which may be less accurate than gastroscopic aspiration, CT, or MRI. Although real-time ultrasound monitoring was used to ensure complete aspiration, more accurate methods should be used. Finally, while our model outperforms others in predicting high GRV, its accuracy decreases when GRV exceeds 3∼4 mL/kg. Future research should include more data on elevated GRV to improve predictive accuracy.

## 6. Conclusion

This study provides support for the use of ultrasound monitoring for GRV in critically ill children. The predictive model we developed can estimate GRV in critically ill children receiving EN and helps identify those with high GRV. In addition, the three-point qualitative grading system is applicable to this population. Further validation in a larger cohort of critically ill children is required before these findings can guide clinical decision-making.

## Figures and Tables

**Table 1:** Subject characteristics.

Variable	Value
Sex (*n*, %)	
Male	68 (63.0%)
Female	40 (37.0%)
Age (years, IQR)	2.62 (0.55, 6.87)
< 1 year old (*n*, %)	35 (32.4%)
1–3 years old (*n*, %)	22 (20.4%)
3–6 years old (*n*, %)	18 (16.7%)
6–12 years old (*n*, %)	16 (14.8%)
12–18 years old (*n*, %)	17 (15.7%)
Weight (kg, IQR)	13 (6.90, 25.00)
Height (m, IQR)	0.90 (0.66, 1.28)
BMI (IQR)	15.87 (14.38, 18.25)
Qualitative grading system scores (*n*, %)	
Grade 0	37 (34.3%)
Grade 1	24 (22.2%)
Grade 2	47 (43.5%)
Main diagnosis (*n*, %)	
Respiratory disease	40 (37.0%)
Craniocerebral injury	31 (28.7%)
Circulatory disease	6 (5.6%)
Neurological disease	4 (3.7%)
Shock	3 (2.8%)
Vascular malformation	2 (1.9%)
Others	21 (19.4%)

*Note:* mL, milliliter; kg, kilogram.

Abbreviation: IQR, interquartile range.

**Table 2:** Antral cross-sectional area and suctioned gastric residual volume according to the qualitative grading system.

Variable	Grade 0 (*n* = 37)	Grade 1 (*n* = 24)	Grade 2 (*n* = 47)	*p* value
Suctioned GRV (mL)	0 (0, 3.50)	13.0 (5.5, 19.5)	37.00 (25.00, 65.00)	< 0.001
Suctioned GRV (mL/kg)	0 (0, 0.26)	1.29 (0.51, 2.28)	2.22 (1.56, 4.05)	< 0.001
Supine CSA (cm^2^)	0.95 (0.75, 1.36)	1.07 (0.88, 1.60)	2.49 (1.36, 3.68)	< 0.001
RLD CSA (cm^2^)	1.27 (0.96, 1.76)	1.98 (1.45, 2.64)	4.55 (2.91, 6.25)	< 0.001

*Note:* mL, milliliter; mL/kg, milliliters per kilogram; cm^2^, square centimeter.

**Table 3:** Regression model statistics for the suctioned gastric residual volume.

Variable	Model 1				Model 2			
	*B*	SE	*t*	*p* value	*B*	SE	*t*	*p* value
Constant	−13.06	2.47	−5.28	< 0.001	−12.91	2.32	−5.55	< 0.001
RLD CSA (cm^2^)	10.25	0.51	20.02	< 0.001	10.29	0.45	22.63	< 0.001
Grade 1	3.36	3.63	0.92	0.368	3.29	3.59	0.91	0.36
Grade 2	10.06	3.41	2.95	0.004	10.06	3.39	2.96	2.96
Age	0.06	0.33	0.18	0.85				
Adjusted *R*^2^	0.877				0.878			
AIC	875.30				873.43			
BIC	888.71				884.06			

*Note:R* ^2^, coefficient of determination.

Abbreviations: AIC, Akaike information criterion; BIC, Bayesian information criterion; CSA, cross-sectional area; RLD, right lateral decubitus; SE, standard error.

## Data Availability

The data that support the findings of this study are available on request from the corresponding author. The data are not publicly available due to privacy or ethical restrictions.

## Supporting Information

Supporting File.

Figure Supporting 1: Correlation analysis between age, weight, height, BMI, supine CSA, RLD CSA, and qualitative grading system scores with GRV; BMI, body mass index; CSA, cross-sectional area; RLD, right lateral decubitus; GRV, gastric residual volume; and Grade, qualitative grading system scores.

Figure Supporting 2: Predicted gastric residual volume as compared to the suctioned gastric residual volume (mL/kg).
