# Supplementary material for: Prediction of Gastric Residual Volume by Ultrasonography in Critically Ill Children Undergoing Enteral Nutrition
Source: Crit Care Res Pract. 2025 Jun 23;2025:1049746. doi: 10.1155/ccrp/1049746 (PMC12208764; doi:10.1155/ccrp/1049746)
Supplement: Supporting Information — Additional supporting information can be found online in the Supporting Information section. [file 1049746.f1.zip › 5-Supplementary File.docx]

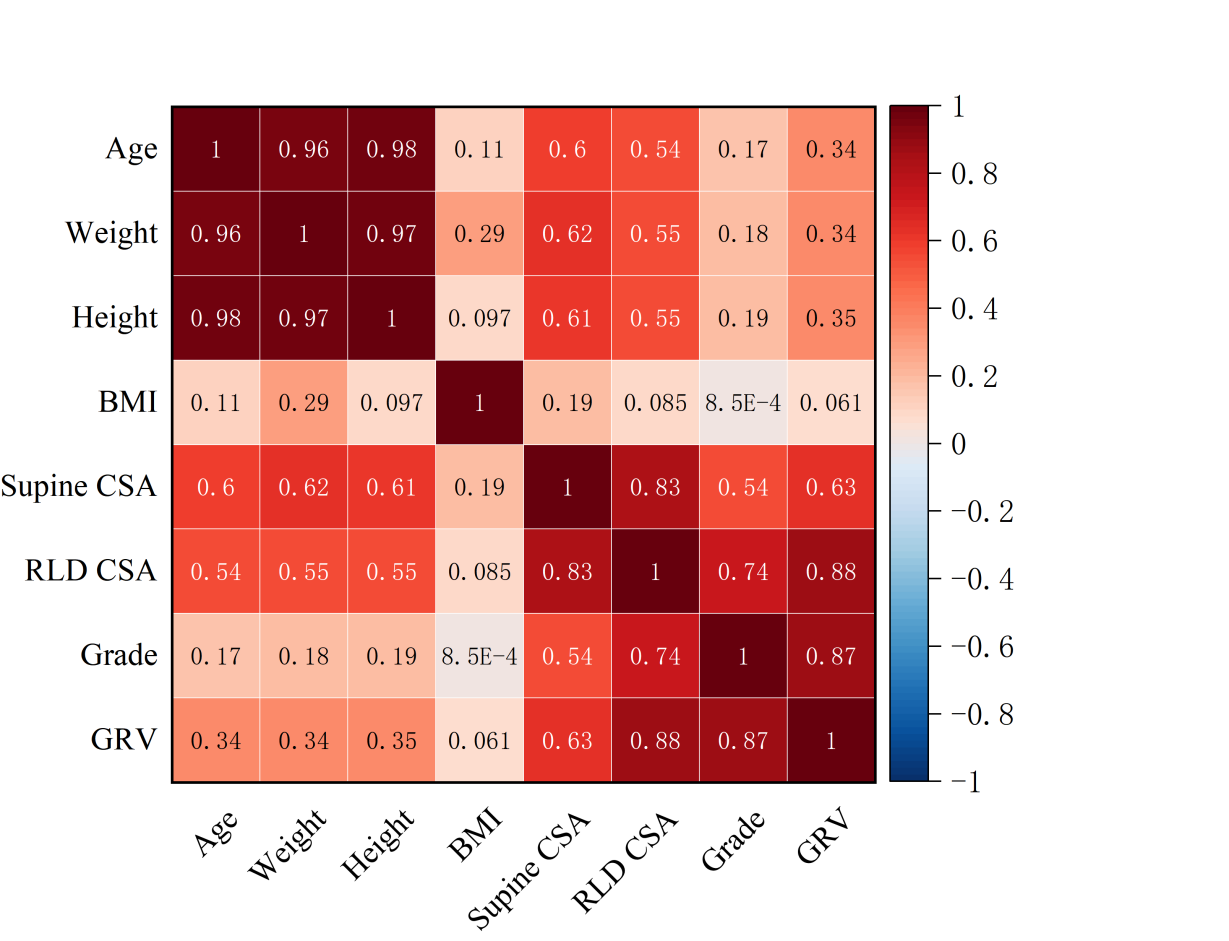


**Figure Supplement 1 C**orrelation analysis between age, weight, height, BMI, supine CSA, RLD CSA, and qualitative grading system scores with GRV; BMI, body mass index; CSA, cross-sectional area; RLD, right lateral decubitus; GRV, gastric residual volume; Grade, qualitative grading system scores.


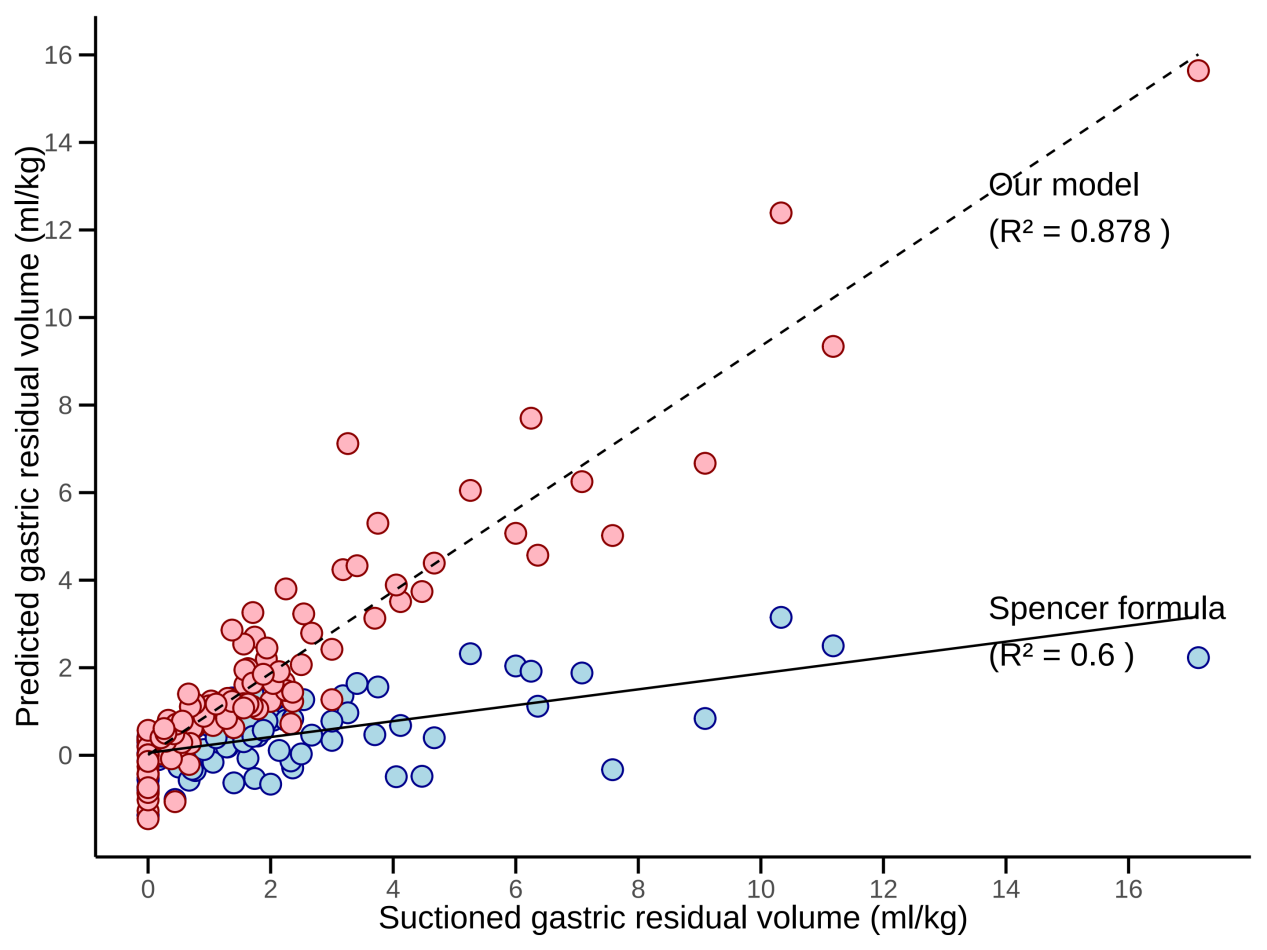


**Figure Supplement 2**. Predicted gastric residual volume as compared with the suctioned gastric residual volume (ml/kg).
