# Supplementary figures and images for: Prediction of Gastric Residual Volume by Ultrasonography in Critically Ill Children Undergoing Enteral Nutrition
Source: Crit Care Res Pract. 2025 Jun 23;2025:1049746. doi: 10.1155/ccrp/1049746 (PMC12208764; doi:10.1155/ccrp/1049746)

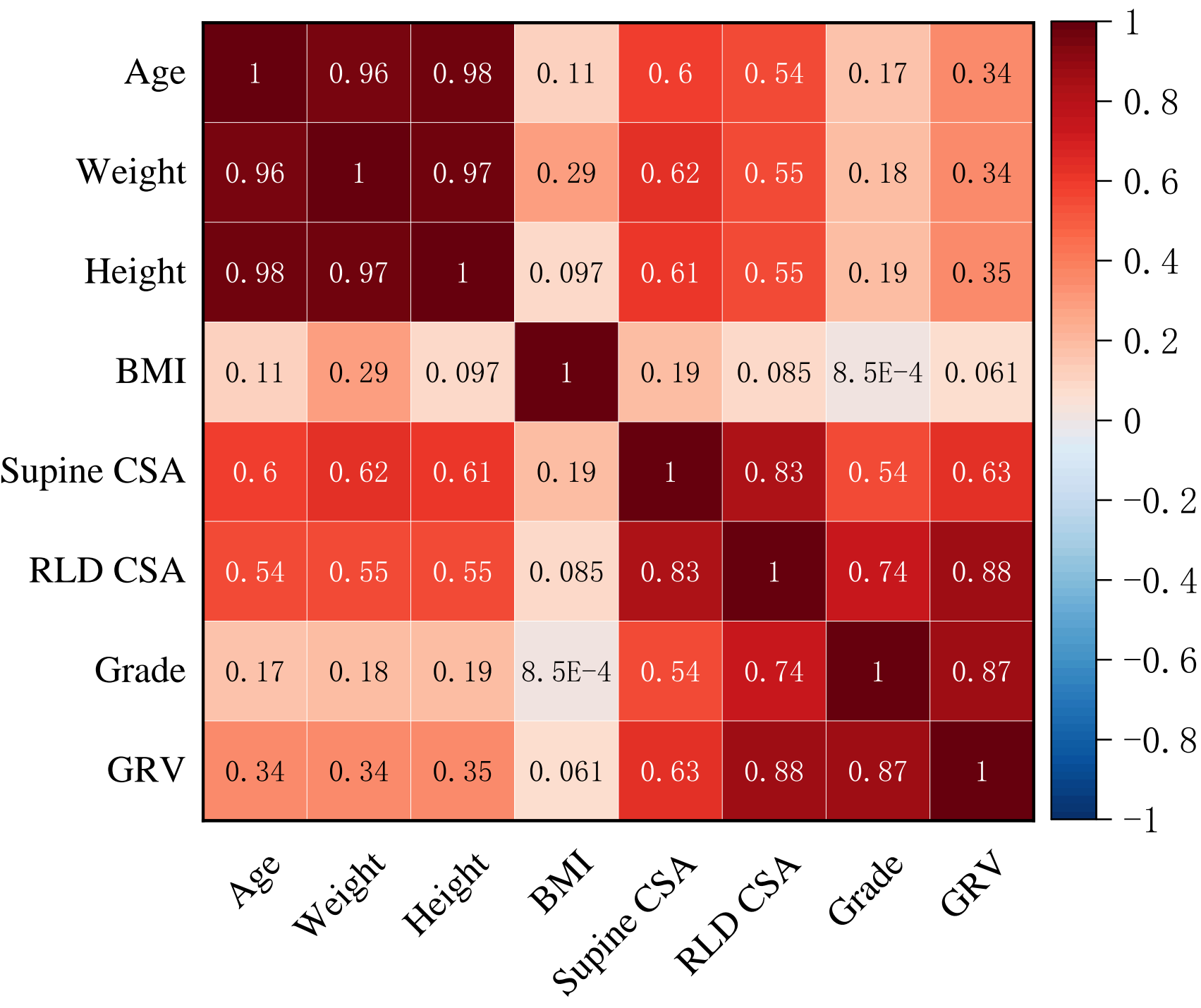

Supplement: Supporting Information — Additional supporting information can be found online in the Supporting Information section. [file 1049746.f1.zip › Figure Supplement 1 (editable).pdf]

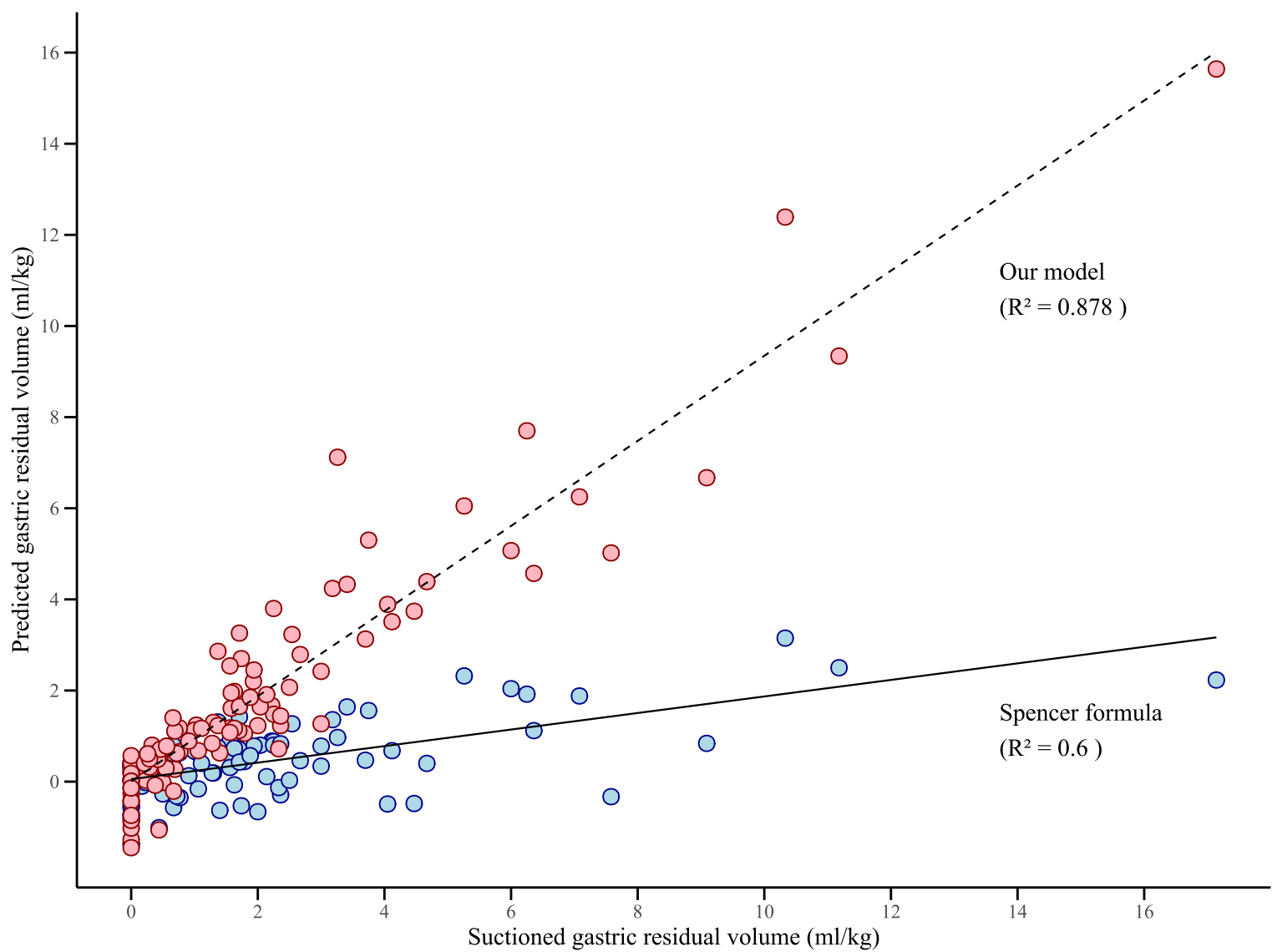

Supplement: Supporting Information — Additional supporting information can be found online in the Supporting Information section. [file 1049746.f1.zip › Figure Supplement 2(editable).pdf]
